# An Intelligent Wireless System for Field Ecology Monitoring and Forest Fire Warning

**DOI:** 10.3390/s18124457

**Published:** 2018-12-16

**Authors:** Yili Zheng, Yandong Zhao, Weiping Liu, Shengbo Liu, Ruting Yao

**Affiliations:** School of Technology, Beijing Forestry University, Beijing 100083, China; yandongzh@bjfu.edu.cn (Y.Z.); hfpl916@126.com (W.L.); podolski@bjfu.edu.cn (S.L.); Yaoruting@bjfu.edu.cn (R.Y.)

**Keywords:** Internet-of-Things, intelligent wireless monitoring, Beidou satellite, forest fire warning

## Abstract

Based on Internet-of-Things and multi-sensor technology, an intelligent wireless monitoring system was developed to obtain field ecological parameters and provide forest fire warning in real-time. The GPRS and China’s Beidou satellite communication were selectively used for date transmission in the field with weak cell phone signals. This monitoring system is mainly composed of several field ecological monitoring stations, a cloud server, and online system software. Atmosphere, soil, sunlight and plant parameters of different regions are obtained real-time by sensors stably and reliably. This system has functions such as field ecological data storage, dynamic query, report generation, and data analysis. As an example of typical application, the forest fire weather grade, which was supplemented with the litter layer soil humidity, was calculated to realize the early warning of the local forest fire in this system through continuous experiments at Beijing Jiufeng National Forest Park from March to May 2017 and Inner Mongolia from March to June 2018. The success ratios of data transmission through Beidou satellite were 98.57%, 99.43%, 99.59%, and 98.85%, respectively, in Beijing, and through GPRS were 99.89% and 99.90% in Inner Mongolia. Long-term real-time field ecological monitoring and forest fire warning were successfully realized. This system can be widely used for big data field acquisition and analysis in forest and agriculture regions.

## 1. Introduction

The real-time continuous monitoring of field ecological system parameters can generate massive big data information sources to reveal the relationships between various ecological factors and their internal variation rules, support ecological conservation actions, and prevent ecological disasters [1].

Currently, the methods for obtaining the parameters for monitoring field ecology, such as atmosphere, soil, light, and plants, fall into four classes. Class 1 consists in consulting the official website of the National Meteorological Observatory for meteorological data. However, the terrain, altitude, and vegetation conditions in a forestry vary substantially, and the unique microclimate environment significantly deviates from the data on the website for the National Meteorological Observatory, thus making it impossible to guarantee the timeliness and accuracy of monitoring data [2,3,4]. Class 2 consists in monitoring the meteorological data or spectroscopic data in a forest region using a meteorological satellite or remote sensing satellite [5,6,7]. This method can only achieve macroscopic wide-range monitoring and early warning of the ecological changes and disasters in forests and cannot obtain fine microscopic data. Class 3 consists in deploying a large number of wireless sensor nodes in a field region [8,9,10,11,12,13]. Sensor nodes can collect microclimate data and send them to their respective cluster nodes by wireless sensor networks. The wireless sensor networks in the field face obstacles such as limited power resources and a high vulnerability to harsh environment. Class 4 consists in building designated ecological monitoring stations in the forest region [14,15,16] to automatically measure the ecological microenvironment data of farmland and forests. This method is widely used because of its advantages, such as high accuracy, saving of labor, and capacity to continuously monitor for a long time. Many ground-based ecological monitoring stations are built to collect the visual or infrared images and microclimate data for forest fire warnings [17,18,19]. Based on static and dynamic analysis of chromatic changes, histogram changes, or luminance changes in images, forest fires can be detected and classified. Different sensor data are used to reduce or eliminate the false alarm rate. In some remote regions or regions without mobile phone signal, this method cannot achieve an effective real-time transmission of data and images, which becomes a bottleneck for its actual application in monitoring field ecology.

The Beidou Satellite, developed by China, is a global satellite navigation system capable of providing positioning and navigation, two-way short message communication, and precise timing services at any location and at any time within the coverage area, thus effectively solving the problem of data communication in remote regions and regions with no or weak mobile phone signal [20,21,22].

Based on the communication of China’s Beidou Satellite and on GPRS communication, this paper develops a real-time monitoring network system for field ecological parameters and achieves real-time monitoring of various parameters such as atmosphere, soil, light, and plants of the forest as well as remote transmission, cloud storage, and query of data. The system serves the acquisition and analysis of ecological big data in remote regions and regions with strong or weak mobile phone signal.

## 2. Design of the Monitoring System

### 2.1. System Composition

**Figure 1:**
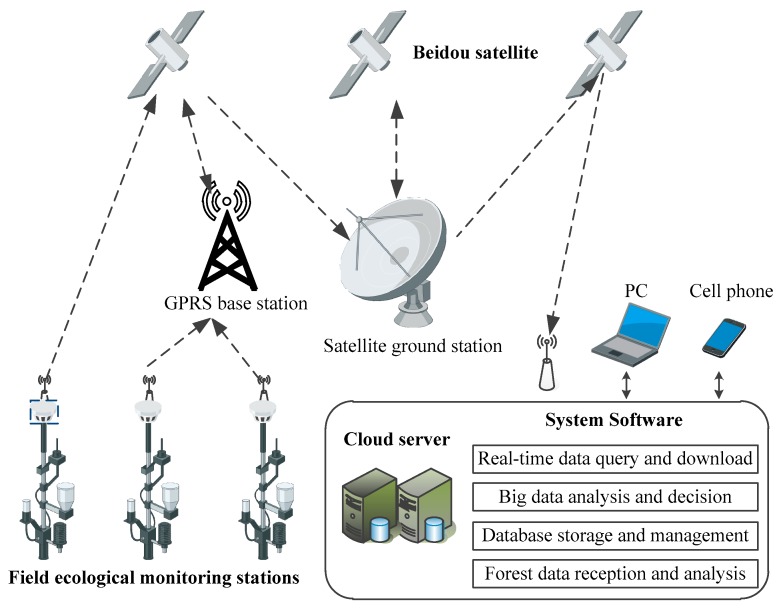
Schematic diagram of the field monitoring system.

The system comprises multiple field ecological monitoring stations, a cloud server, and online system software, as shown in Figure 1.

The field ecological monitoring station is connected with the sensor groups of atmosphere, soil, light, and plant parameters and is driven by solar energy. Two-way communication occurs between the field ecological monitoring station and the cloud server via the short message communication module of the Beidou satellite or GPRS. The system software runs on the cloud server provided by the Alibaba Cloud with functions such as real-time data query and download, big data analysis and decision-making, database storage and management, satellite data reception, and parsing. Multiple field ecological monitoring stations are networked for all-round monitoring of the microenvironment in forest regions. Users may inquire and download the ecology monitoring data of forest regions using a PC or a mobile phone via remote network connection.

The field ecological monitoring stations automatically acquire data from the connected sensors for atmosphere, soil, light, and plants at an interval of 10 min, manually adjustable, according to their own clocks. In the forest region with weak mobile phone signal, the data are stored and reformatted to form a 72-byte short message communication package and sent to the Beidou Satellite through the communication module of the Beidou Satellite. The ground station of the Beidou Satellite receives the satellite data and in return sends the data to the cloud server through the Beidou Satellite. In the forest region with a stable mobile phone signal, GPRS is used for data transmission.

The system software can receive and parse the communication package of the Beidou satellite or GPRS, and then store the monitoring data into the MySQL database relying on its functions of receiving and parsing the satellite data. The real-time data query and download function allows for real-time access to the database. PC and mobile phone users can enjoy the services, such as remote data access, curve query, and statement download, through the Internet or the mobile communication network. The database storage and management function allows for operations of the data in the MySQL database, such as addition, deletion, modification, updating, and search.

The big data analysis and decision-making function of the system software allows for a determination of the grade of fire risk weather in forest regions, prediction and early warning of fire disasters in forests, an evaluation of the ecological environment restoration, decision-making on accurate irrigation, monitoring of growth state of stumpage, etc., according to the microclimate monitoring data acquired and the national standards or big data analysis models.

### 2.2. Design of Online System Hardware

**Figure 2:**
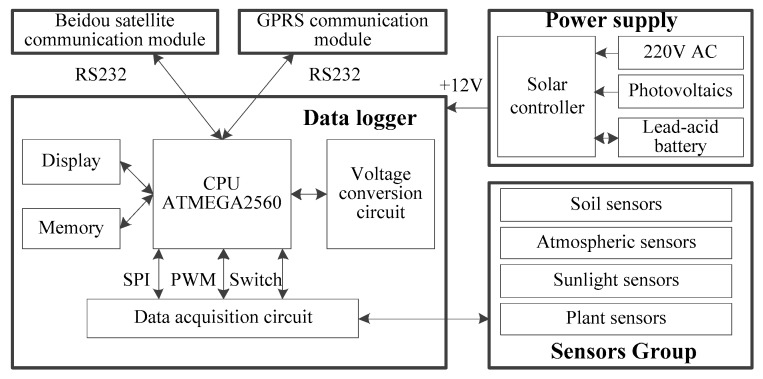
Block diagram of the system hardware.

The hardware of the field ecological monitoring station in forest regions is comprised of data acquisition units, a power supply system, sensor groups, Beidou Satellite communication modules, GPRS communication modules, and mechanical towers, as shown in Figure 2.

The data acquisition unit is the data processing and control core of the whole field ecological monitoring station, composed of a central processing unit, a display circuit, a storage circuit, a data acquisition circuit, etc. The data acquisition unit can acquire the analog or digital signal output by each sensor and complete data transmission through the Beidou Satellite as required by users.

The central processing unit of the data acquisition unit uses the ATMEGA2560 single chip microcomputer of the ATMEL Company. The central processing unit completes the parsing of the original data from each sensor, the conversion of the data communication format, the conversion of the data display format, the conversion of the data storage format, the monitoring of the sensor operation mode, the monitoring of the satellite and GPRS communication mode, and the monitoring of the power supply mode and achieves automatic reset following operator errors via the watchdog circuit. A color monitor is used for displaying the monitoring data and status data. The data storage circuit uses an SD card with a storage capacity of 8 G for locally storing monitoring data, allowing for manual data copying and erasing at regular intervals. The data acquisition circuit uniformly converts a 0–20 mA analog current signal and a 0–5 V analog voltage signal output, using the sensor group, into a 0–2.5 V analog voltage signal and is connected to the AD conversion interface of the single chip microcomputer after amplification and filtering. The IO interface of ATMEGA2560 acquires the switching value output via the precipitation sensor. The PWM interface acquires the output of the wind speed sensor.

The communication module of the Beidou Satellite uses the GYT2015C Beidou Satellite data transmission module. The data transmission module has the Beidou Satellite communication and Beidou Satellite positioning and timing functions and is connected to the central processing unit via the RS232 interface, with a transmitting power of 10 W. The communication module of the Beidou Satellite is installed on the mechanical rack for providing the communication link of the Beidou Satellite for the field ecological monitoring station. The system reserves a GPRS communication module interface for use in the regions with mobile phone signal.

The sensor group can measure the parameters of soil, atmosphere, light, and plants in the ecological microenvironment in forest regions in real time. The major sensor configuration and properties are shown in Table 1. Users may increase or decrease the number of sensors as necessary.

The power supply system is comprised of a solar energy and controller, a 75 Watts polycrystalline solar panel, and a 12 V 65 A·h gel energy storage battery and provides a 12 V direct current for the field ecological monitoring stations. A 220 V alternating current power supply can be used for the forest where mains supply is available. The energy storage battery is placed in an underground water-proof box and buried 0.5 m below the soil surface under the mechanical tower for anti-freezing protection.

The mechanical tower is made of stainless steel, fixed with stainless steel wire ropes for wind protection, and provided with a lightning conductor, with its foundation fixed by heavy concrete blocks. The mechanical tower is equipped with atmosphere parameter sensor groups, light parameter sensor groups, Beidou satellite and GPRS data transmission modules, and solar energy panels at the top. The mechanical tower is equipped with a stainless-steel rain-proof box in the middle and other components inside it, such as data acquisition unit and a solar energy controller. The soil parameter sensor group includes multiple soil moisture and temperature sensors, which may be buried in the soil at different depths in different positions for measuring the temperatures and moistures of the soil in forests. In terms of plant parameters, a non-destructive packaged stumpage stem moisture sensor and a needle-type stumpage stem moisture sensor are used to measure the volumetric moisture of stumpage stems with a diameter at breast height smaller than 10 cm and a diameter at breast height greater than 10 cm, respectively. 

**Figure 3:**
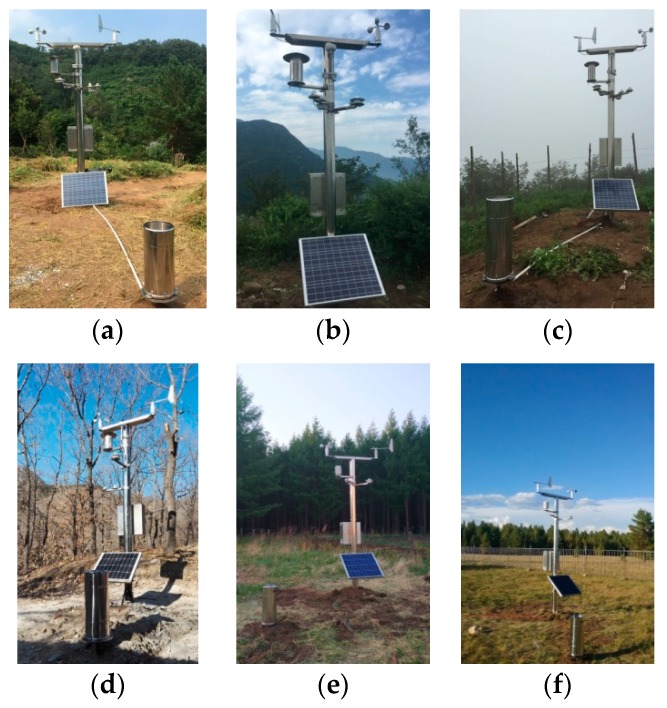
Forest microclimate monitoring stations in Beijing and Inner Mongolia. (**a**) Yan’er Ridge; (**b**) Wangjing Tower; (**c**) Luobadi; (**d**) Zhai’er Yu; (**e**) Hulun Buir; (**f**) Zhalantun.

**Figure 4:**
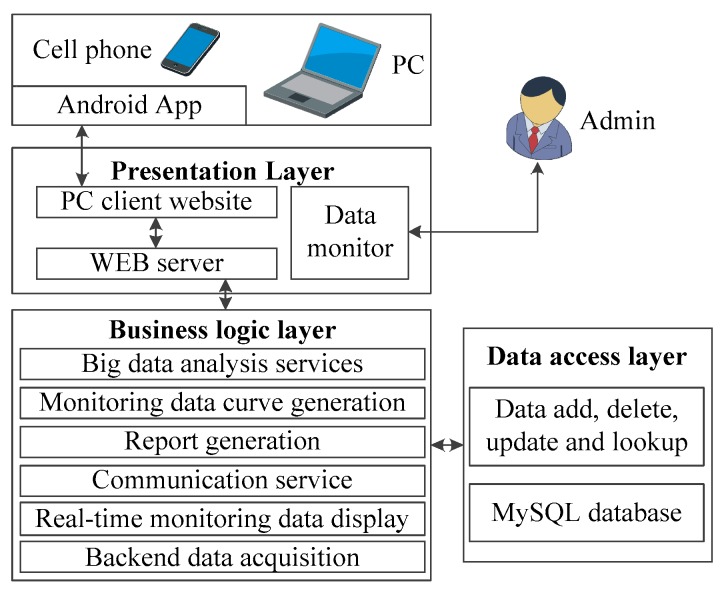
Block diagram of the software.

Figure 3a–d presents the three ecological monitoring stations using Beidou satellite communication located on Yan’er Ridge (at an altitude of 440 m), Wangjing Tower (at an altitude of 750 m), and Luobadi Peak (at an altitude of 1000 m) in Beijing Jiufeng National Forest Park. No stable GPRS communication can be achieved for cell phones, as this region is far from the mobile base station and affected by the air route. Figure 4e–f present the two ecological monitoring stations using GPRS communication located on and Hulun Buir and Zhalantun in Inner Mongolia. No stable Beidou satellite short message communication can be achieved, as this region is far from the geosynchronous orbit of the Beidou satellite.

### 2.3. Design of System Software

The system software runs continuously on the Alibaba cloud server. The overall framework is constructed based on the. NET technology, including the front-end presentation layer, the business logic layer, and the data access layer. Figure 4 presents the overall architecture of the system software.

The presentation layer is responsible for interactions between the system and multiple concurrent users. The business logic layer deals with the core businesses, such as monitoring data reception, big data analysis, and the logical processing of data to meet the requirements of the presentation layer and the monitoring tasks. The data access layer directly operates the database for addition, deletion, modification, updating, and search of the monitoring data.

The PC front-end website of the presentation layer is designed with HTML, CSS, and the JavaScript scripting language. The visualized chart database Echarts.JS of Baidu is used to design the charts, dashboards, and dynamic components [23] for the monitoring data. In terms of data monitoring program, the Winform technique is used for setting up web pages. The administrators can monitor the operating state of the communication between the sensor and the Beidou Satellite. The WEB server program uses IIS of Microsoft and exchanges information with users’ PC browsers via HTTP (hypertext transmission protocol).

The Java language is used to develop the Android application for mobile phones. The HTTP protocol is used to upload and download data to and from the cloud server. The MPAndroidChart open-source framework is used to achieve the query and display of real-time data on forest ecology, variation curve, grade of fire weather, etc.

The communication service program receives the monitoring data and operative mode data transmitted by the field ecological monitoring station via the short message communication of the Beidou Satellite or GPRS. The monitoring data generation program is used to generate daily statements, monthly statements, and annual statements of monitoring data in Excel format. The monitoring data curve program is used to generate daily variation, monthly variation, and annual variation curves for various ecological parameters. The real-time data generation program provides real-time varying data for the dashboard controls of the PC front-end website. The big data analysis service program can achieve evaluation of the ecological environment restoration, making decisions on accurate irrigation, the monitoring of the stumpage growth state, etc. The back-end data acquisition program is connected with the data monitoring program of the presentation layers to complete the parameter setting for the data acquisition unit, the parsing of data from various sensors, format conversion, and the automatic processing of errors and missing data.

The data access layer uses the MySQL database of open source code to provide storage space for all data and utilizes the structured query language SQL for database management.

The system software uses Load Runner for testing, supports 1200 concurrent users, and reserves interfaces for subsequent application and extension; it therefore meets requirements such as real-time capability, multiple concurrence, and extendibility.

**Figure 5:**
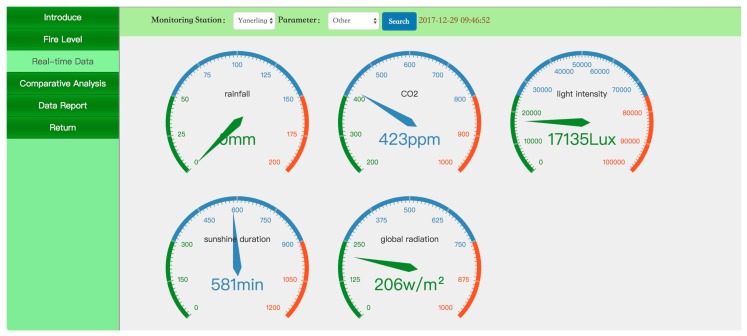
Real-time monitoring data of the forest microclimate.

**Figure 6:**
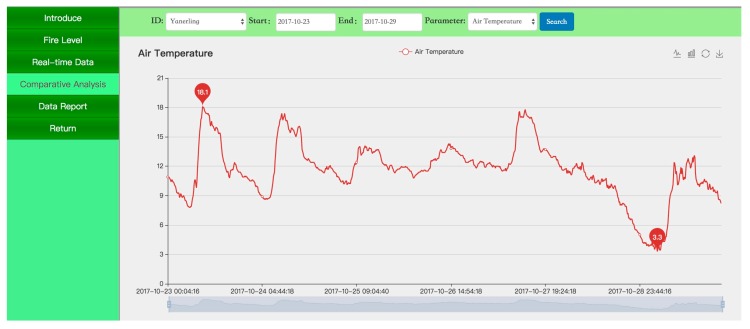
Air temperature curve of the forest microclimate.

**Figure 7:**
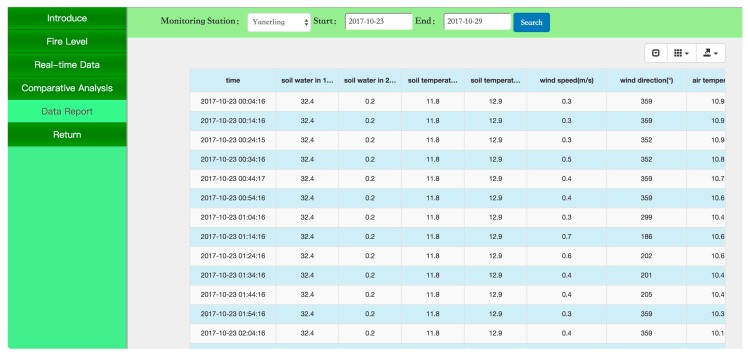
Monitoring data reports of the forest microclimate.

Figure 5, Figure 6 and Figure 7 present screenshots of the PC website. Figure 5 shows the real-time data on parameters such as temperature, moisture, and precipitation in the forest microenvironment in the form of a dashboard. Figure 6 shows the changes in forest temperature in one week in the form of a curve. Figure 7 shows ecological monitoring data of the forest microenvironment in the form of a statement. The data statements can be exported in Excel format.

## 3. Prediction of the Fire Weather Grade

The fire weather grade is an important index for measuring the possibility of forest fire occurrence and the difficulty level of its spread. Additionally, it is an important basis for forest fire protection management. The forecasts made by the Bureau of Meteorology are based on macroscopic data and are not specific enough to predict the grade of forest fire risk under different altitude, vegetation, or microclimate conditions. The microenvironment monitoring system for forest ecology can acquire and compute the local meteorological factors required for forecasting the grade of forest fire risk in real time, which plays an important role in improving the fire weather forecast and correct decision making.

According to China’s Fire Weather Grades released by Forestry and Grassland Administration in 1995, the assessment indexes of fire weather grades are comprised of the daily highest air temperature *A*, the daily lowest relative humidity *B*, the number of consecutive days without precipitation *C*, the daily maximum wind scale *D*, phenology correction index *E*5, and partial fire weather indexes. Indexes *A*, *B*, *C*, and *D* are determined using meteorological monitoring parameters. Index *E* is determined based on the empirical value or monitoring value of phenological changes and substituted into the following equation [24]:

(1) A+B+C+D−E.

The standard is used to compute the fire weather grade. Scores are given according to the stepwise intervals. Fire risk grades with major differences are computed based on the meteorological data with minor differences. However, Correction Index *E* does not consider the soil moisture conditions of forests.

The system computes the fire weather index based on real-time data obtained from the field ecological monitoring station. The computation of fire weather index is smoothed using the logistic regression function [25]. The litter layer soil moisture is introduced for index correction.

### 3.1. Smoothing of the Computation of the Fire Weather Index

According to the industrial standard, the median *X_i_* (*i* = *A*, *B*, *C*, *D*) scored in stepped intervals of *A*, *B*, *C*, and *D* and the corresponding fire weather index *Y_i_* constitute a fitted sample set.

The fitted sample set of Index *A* is

{*X_A_*, *Y_A_*} = {(2.5, 0), (7.5, 4), (12.5, 8), (17.5, 12), (22.5, 16), (27.5, 20)}.

The fitted sample set of Index *B* is 

{*X_B_*, *Y_B_*} = {(75%, 0), (65%, 4), (55%, 8), (45%, 12), (35%, 16), (25%, 20)}.

The fitted sample set of Index *C* is

{*X_C_*, *Y_C_*} = {(0.3, 10), (0.9, 8), (2.0, 6), (3.5, 4), (5, 2), (7.5, 0)}.

The fitted sample set of index *D* is

{*X_D_*, *Y_D_*} = {(0.1, 0), (0.9, 5), (2.5, 10), (6.7, 15), (9.4, 20), (12.3, 25), (15.5, 30), (19.0, 35), (20.7, 40)}.

The logistic function

(2) $$Y_{i}^{\prime }=\frac{K_{i}}{1+e^{\alpha_{i}+\beta_{i}X_{i}}}$$

is used for fitting the sample. Here, *K_i_* is determined by the industrial standard, *K_i_* = {20, 20, 10, 40}, and *α_i_* and *β_i_* are the fitted weights. By fitting based on Equation (2), the computation functions of *A*, *B*, *C*, and *D* are

(3) $$Y_{A}^{\prime }=\frac{20}{1+e^{3.6421-0.2428X_{A}}}$$

(4) $$Y_{B}^{\prime }=\frac{20}{1+e^{-6.0701+12.1403X_{B}}}$$

(5) $$Y_{C}^{\prime }=\{\begin{array}{c} \frac{10}{1+e^{-2.5545+0.8685X_{C}}}+5t\;\;\;\left(X_{C}\leq 10\right)\\ 5\left(t-1\right)\;\;\;\;(X_{C}>10) \end{array}$$

(6) $$Y_{D}^{\prime }=\frac{40}{1+e^{2.2844-0.2396X_{D}}}.$$

In Equation (5), *t* is the number of consecutive days with precipitation, and the maximum value $Y_{C}^{\prime }$ is 50. The determination coefficients *R*^2^ of fitting indexes *A*, *B*, *C*, and *D* in Equation (3)–(6) are 0.9771, 0.9771, 0.9691, and 0.9656, respectively.

### 3.2. Computation of Fire Weather Grade

The phenological correction index *E* in the industrial standard only considers the degree of coverage. The litter layer soil in forests contains many combustibles. The soil moisture of the litter layer has significant effects on the occurrence and spread of forest fire [26].

In the system, the phenological correction index E is comprised of $Y_{E1}^{\prime }$ (the degree of coverage) and $Y_{E2}^{\prime }$ (the litter layer soil moisture index); $Y_{E1}^{\prime }$ is obtained on the basis of the statistical empirical value, while $Y_{E1}^{\prime }$ can be increased to 15 in the fire season and to 20 in the core fire season, and can be decreased to 0 beyond the fire season. The litter layer is comprised of a undecomposed layer, a semi-decomposed layer, and a humus layer in a top-down order. The soil moisture sensor can directly measure the volumetric water content of the humus layer and determine the size of $Y_{E2}^{\prime }$.

There are significant differences in the moisture content of the litter layer soil between different tree species. According to the laboratory test, the volumetric moisture content of the cork oak humus layer ranges between 4.2 and 97.6% [27]. $Y_{E2}^{\prime }$ is set to –20 when the volumetric moisture content of the humus layer is above 30%. $Y_{E2}^{\prime }$ is set to 0 when the volumetric moisture content of the humus layer is below 5%. $Y_{E2}^{\prime }$ and the volumetric moisture content of the humus layer exhibit a linear relationship when the volumetric moisture content of the humus layer is between 5% and 30%. By linear fitting, we can obtain the computation function of Index $Y_{E2}^{\prime }$:

(7) $$Y_{E2}^{\prime }=\{\begin{array}{c} 0\\ 4-80X_{E2}\;\\ -20 \end{array}\begin{array}{c} \left(X_{E2}<5\%\right)\\ \left(5\%\leq X_{E2}<30\%\right)\\ \left(X_{E2}\geq 30\%\right) \end{array}.$$

The air temperature, air moisture, precipitation, wind force, and the soil moisture at a depth of 10 cm obtained from the field ecological monitoring station are substituted into Equations (3)–(7) in sequence to obtain $Y_{i}^{\prime }$. The corrected index of fire weather grade is

(8) $$Y_{A}^{\prime }+Y_{B}^{\prime }+Y_{C}^{\prime }+Y_{D}^{\prime }+Y_{E1}^{\prime }+Y_{E2}^{\prime }.$$

In the system, the fire weather grade is determined by Equation (8). The correspondence relationship is shown in Table 2 [24].

**Figure 8:**
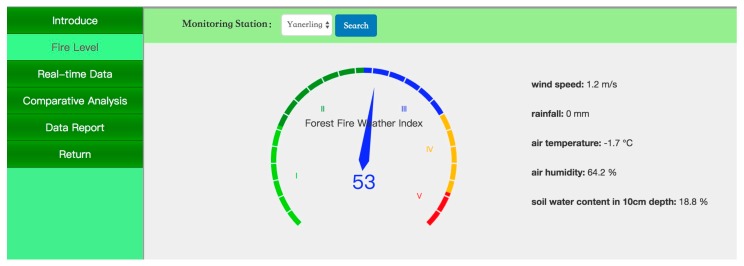
Local forest fire weather grade under the forest microclimate.

Figure 8 presents the grade of fire weather in the forest microclimate displayed on the PC website in real time, which is updated at an interval of 10 min in the form of a dashboard.

## 4. Test and Analysis of the Microenvironment Monitoring System for Forest Ecology

### 4.1. Testing of System Stability

To verify the stability of the system operation, the data of 92 consecutive days obtained from the four field ecological monitoring stations located in Beijing Jiufeng National Forest Park from 1 March 2017 to 31 May 2017 and two monitoring stations located in Inner Mongolia from 31 March 2018 to 30 June 2018. The 13 sensors of 11 types include atmosphere sensors (air temperature, air moisture, carbon dioxide concentration, precipitation, wind speed, and wind direction), light sensors (sunshine hours, total radiation, and light intensity), and soil sensors (two-layer soil moisture and two-layer soil temperature). A network is constructed to monitor the forest ecology. The monitoring data is transmitted in frame via the Beidou satellite or GPRS with each frame containing 72 bytes of data.

Table 3 presents the result of data communication at the field ecological monitoring station in forests. A total of 13,247 frames of field ecological data were acquired with six field ecological monitoring stations in 92 days. The cloud server correctly received 13,058, 13,172, 13,194, and 13,094 frames of ecological data from Yan’er Ridge, Wangjing Tower, Luobadi tower, and Zhai’er Yu, respectively, with correct rates of 98.57%, 99.43%, 99.59%, and 98.85% respectively. The cloud server correctly received 13,232 and 13,235 frames of ecological data from Hulun Buir and Zhalantun in Inner Mongolia by GPRS, respectively, with correct rates of 99.89% and 99.90%, respectively. No data loss lasting for more than 30 min and data errors occurred. The correct rate of data transmission is high enough to meet the data communication requirements of the microenvironment monitoring system for forest ecology.

### 4.2. Data Analysis 

Yan’er Ridge and Hulun Buir are two typical sites for the field microclimate monitoring and forest fire warning. The mobile phone signal is unavailable in the Yan’er Ridge site, so Beidou satellite is chosen for the data communication. The mobile phone signal is good in the Hulun Buir site, so GPRS is chosen for data communication. The result of these two sites is given to show the validation of the system function.

**Figure 9:**
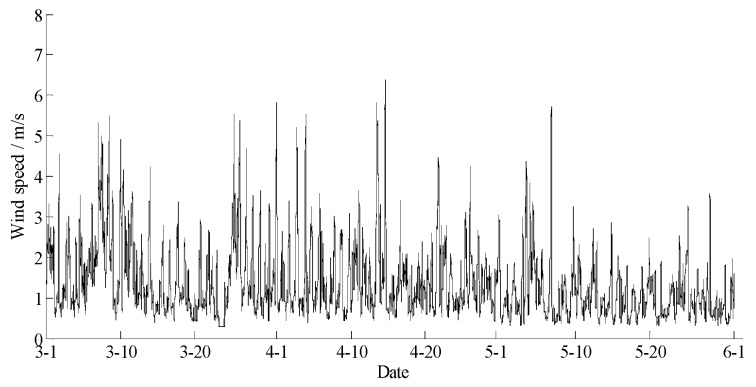
The curve of wind speed in Yan’er Ridge.

**Figure 10:**
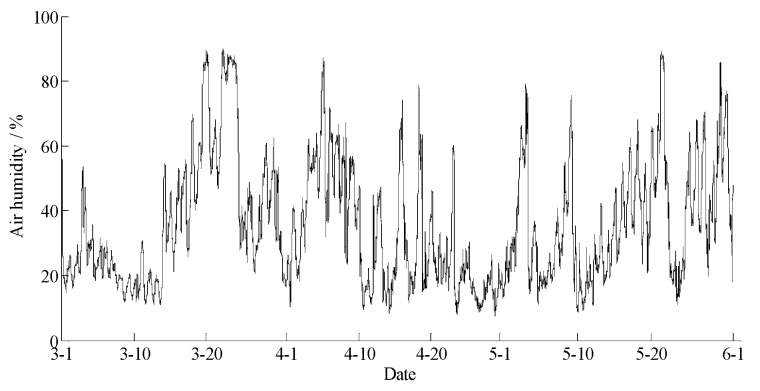
The curve of air humidity in Yan’er Ridge.

**Figure 11:**
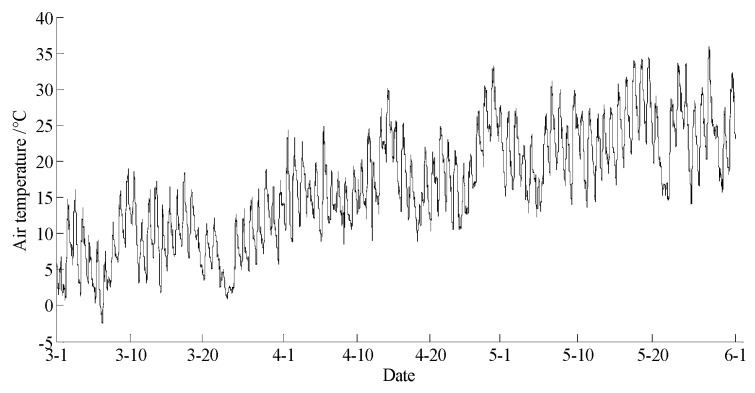
The curve of air temperature in Yan’er Ridge.

**Figure 12:**
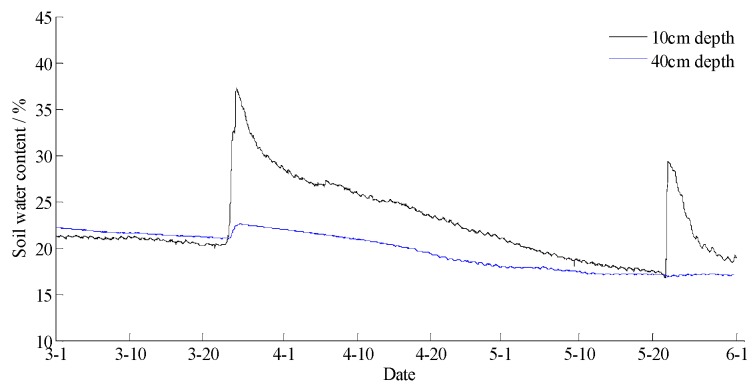
The curves of soil water content in 10 and 40 cm depth in Yan’er Ridge.

**Figure 13:**
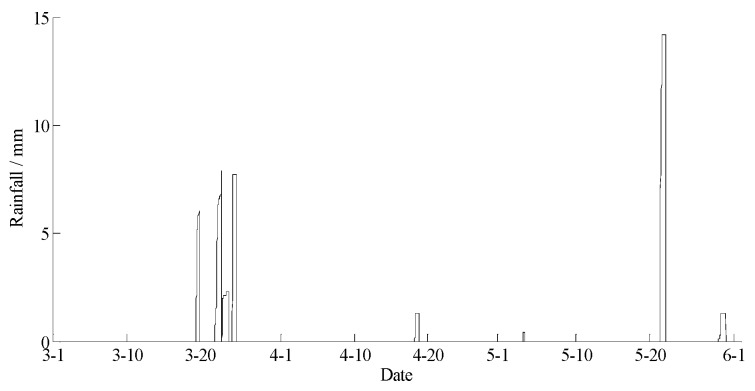
The curve of rainfall in Yan’er Ridge.

**Figure 14:**
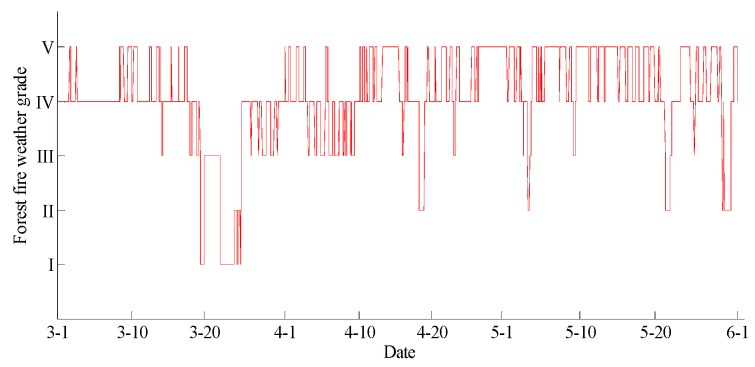
The curve of the forest fire weather grade in Yan’er Ridge.

The data statements of consecutive days from 1 March 2017 to 31 May 2017 in Yan’er Ridge were downloaded from the cloud server. The variation curves for wind speed, air moisture, air temperature, soil moisture at depths of 10 and 40 cm, and precipitation related to forest fire protection are shown in Figure 9, Figure 10, Figure 11, Figure 12 and Figure 13. The grades of fire weather in the Yan’er Ridge region from 1 March 2017 to 31 May 2017 computed by the system are shown in Figure 14.

As shown in Figure 13, continuous precipitation occurred on March 20, 23, and 24. As shown in Figure 14, the moisture delay of soil at depths of 10 and 40 cm rose rapidly. The index of forest fire protection decreased; the soil moisture decreased slowly after the precipitation stopped. When the moderate rain lasted for a brief period on May 22, the moisture of the soil at a depth of 10 cm increased, and the fire protection index decreased. The moisture of the soil at a depth of 40 cm remained unchanged as the sensor was deeply buried and hardly affected by the short-term precipitation that occurred on May 22. The moisture of the soil at depths of 10 and 40 cm remained unchanged on April 19 and May 30 due to little precipitation.

**Figure 15:**
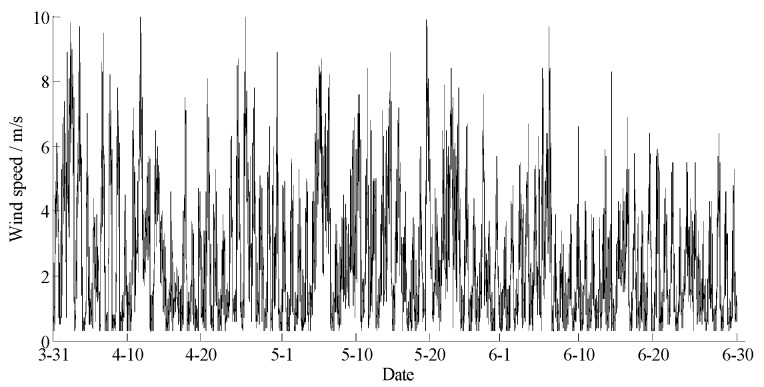
The curve of the wind speed in Hulun Buir.

Based on a comparison between the grade of fire weather in the Yan’er Ridge region in Figure 15 and the variation of precipitation in Figure 14, the fire weather was Grade IV or Grade V in the case of no precipitation in the whole fire protection period, suggesting higher risks of forest fire occurrence and spread. The fire weather was Grade II on April 19, May 22, and May 30 when precipitation lasted for a brief period. From March 20 to 24, when precipitation lasted continuously, the fire weather was Grade I or Grade II, suggesting lower risks of forest fire occurrence and spread and effectively forecasting the fire weather grade in this region.

**Figure 16:**
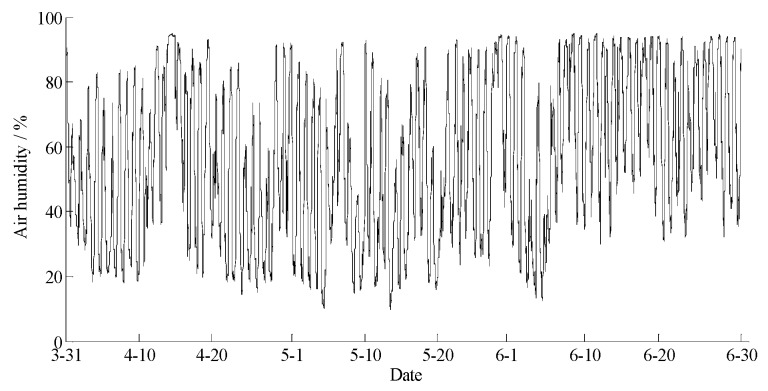
The curve of the air humidity in Hulun Buir.

**Figure 17:**
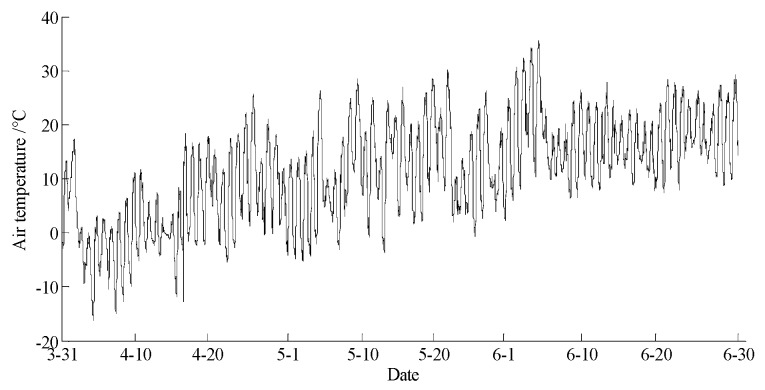
The curve of the air temperature in Hulun Buir.

**Figure 18:**
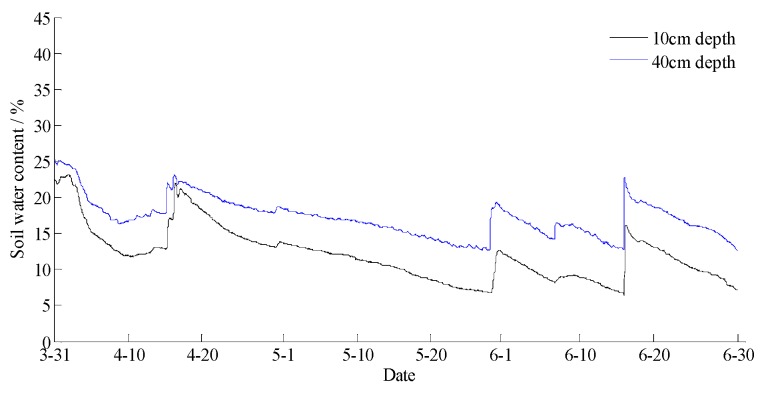
The curves of soil water content at 10 and 40 cm depths in Hulun Buir.

**Figure 19:**
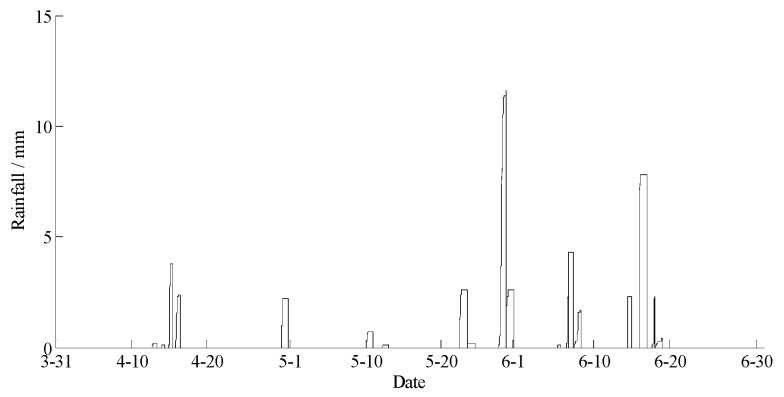
The curve of rainfall in Hulun Buir.

**Figure 20:**
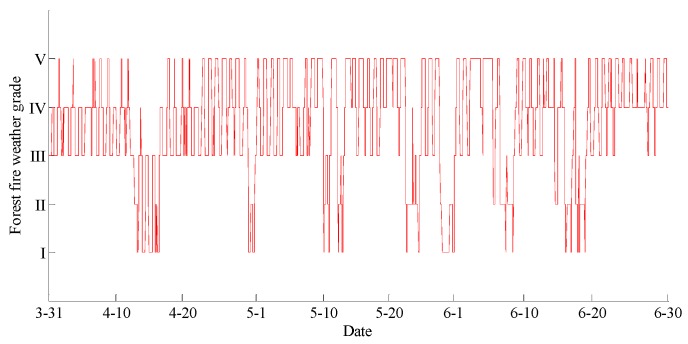
The curve of the forest fire weather grade in Hulun Buir.

The data statements of consecutive days from 31 March 2018 to 30 June 2018 in Hulun Buir were downloaded from the cloud server. The variation curves for wind speed, air moisture, air temperature, soil moisture at depths of 10 and 40 cm, and precipitation related to forest fire protection are shown in Figure 15, Figure 16, Figure 17, Figure 18 and Figure 19. The grades of fire weather in the Hulun Buir computed by the system are shown in Figure 20.

As shown in Figure 19, continuous precipitation occurred on April 15, June 6, and June 16. In Figure 18, the moisture delay of soil at depths of 10 and 40 cm rose rapidly; the index of forest fire protection was decreasing; the soil moisture decreased slowly after the precipitation stopped. Based on a comparison between the grade of fire weather in the Hulun Buir in Figure 20 and the variation of precipitation in Figure 19, the fire weather was Grade III, IV, or V in the case of no precipitation, suggesting higher risks of forest fire occurrence and spread. The fire weather was Grade I or II when precipitation lasted for a brief period, suggesting lower risks of forest fire occurrence and spread and effectively forecasting the fire weather grade in this region.

## 5. Conclusion

In this paper, we presented the design of a field monitoring network system for forest ecology based on the communication functionality of the Beidou satellite and GPRS. This design achieves real-time monitoring, cloud storage, dynamic query, statement generation, and big data analysis of parameters such as atmosphere, soil, light, and plants. The system solves the step-problem in quantitative scoring of the interval of fire weather indexes using the logistic regression function and achieves a fine forecasting of fire weather grades by introducing the factor of litter layer soil moisture. During the experiment conducted at the Beijing Jiufeng National Forest Park and Inner Mongolia, the system could stably and reliably obtain 11 types of microclimate data of forest with a correct rate of transmission of over 98.57% by the Beidou satellite and over 99.89% by GPRS. The system can be used for the big data monitoring of field ecology, an assessment of ecological restoration, meteorological disaster monitoring, accurate irrigation, etc.

## Figures and Tables

**Table 1:** Sensor configuration and performance of the forest microclimate monitoring station.

Group	Sensor Type	Range
soil parameters	soil temperature (2–6 layers)	−55–+80°C
	soil moisture (2–6 layers)	0–100%
air parameters	air temperature	−55–+80°C
	air moisture	0–100%
	carbon dioxide concentration	400-5000 parts per million
	wind direction	0°–360°
	wind speed	0.3–60 m/s
	precipitation	0–4 mm/min
	negative oxygen ion	0–50,000 Ions/cm^3^
	particulate matter 2.5	0-500 μg/m^3^
light parameters	global radiation	0–2000 W/m^2^
	light intensity	0–200,000 Lux
	sunshine hours	threshold 120 W/m^2^
	photosynthetically active radiation	0–4000 μmol/m^2^/s
plant parameters	needle-type stem moisture	0–100%
	non-destructive stem moisture	0–100%

**Table 2:** Forest fire weather grade form.

Fire Weather Grade	
Grade I	≤25
Grade II	26~50
Grade III	51~72
Grade IV	73~90
Grade V	≥91

**Table 3:** The communication result of forest microclimate station.

Location	Communication Mode	Quantity of Data Acquired /Frame	Quantity of data Correctly Transmitted/Frame	Correct Rate /%
Yan’er Ridge	Beidou	13,247	13,058	98.57
Wangjing Tower	Beidou	13,247	13,172	99.43
Luobadi	Beidou	13,247	13,194	99.59
Zhai’er Yu	Beidou	13,247	13,094	98.85
Hulun Buir	GPRS	13,247	13,232	99.89
Zhalantun	GPRS	13,247	13,235	99.90
